# Phytochemical Volatiles as Potential Bionematicides with Safer Ecotoxicological Properties

**DOI:** 10.3390/toxics12060406

**Published:** 2024-06-03

**Authors:** Tomás Cavaco, Jorge M. S. Faria

**Affiliations:** 1 Instituto Nacional de Investigação Agrária e Veterinária (INIAV, I.P.), Quinta do Marquês, 2780-159 Oeiras, Portugal; tomasfcavaco@gmail.com; 2 Instituto Superior de Agronomia (ISA), Universidade de Lisboa, 1349-107 Lisboa, Portugal; 3 GREEN-IT Bioresources for Sustainability, Instituto de Tecnologia Química e Biológica, Universidade Nova de Lisboa (ITQB NOVA), Av. da República, 2780-157 Oeiras, Portugal

**Keywords:** allelochemicals, bionematicides, environmental modelling, pesticides, phytochemicals, plant-parasitic nematodes, risk assessment

## Abstract

The management of plant-parasitic nematodes (PPNs) still relies on traditional nematicides that threaten the environment and human health. Novel solutions are urgently needed for PPN pest management that are effective while safeguarding non-target organisms. Volatile phytochemicals belong to a structurally diverse group of bioactive metabolites that are believed to hold safer environmental characteristics than synthetic pesticides. Nonetheless, not many studies have analysed the potential environmental benefits of shifting to these novel bionematicides. In the present study, 20 phytochemical volatiles with reported nematicidal activity were compared to traditional pesticides using specific parameters of environmental and human health safety available on applied online databases and predicted in silico through specialised software. Overall, the reviewed nematicidal phytochemicals were reportedly less toxic than synthetic nematicides. They were predicted to disperse to the air and soil environmental compartments and were reported to have a lower toxicity on aquatic organisms. On the contrary, the synthetic nematicides were reportedly toxic to aquatic organisms while showing a predicted high affinity to the water environmental compartment. As alternatives, β-keto or fatty acid derivatives, e.g., aliphatic alcohols or ketones, showed more adequate properties. This study highlights the importance of complementing studies on nematicidal activity with a risk assessment-based analysis to allow for a faster selection of nematicidal phytochemical volatiles and to leverage the development and implementation of bionematicides.

## 1. Introduction

Traditional nematicides have been essential tools in nematode pest management worldwide [1]. However, their misuse endangers the surrounding ecosystems, plants, animal wildlife, and, more directly, soil biodiversity [2,3]. Under these conditions, plant pests can reach large population numbers and develop resistance, increasing the probability of spreading to new locations. Currently, traditional pesticides are still amply used for crop protection despite the increasing societal and political pressures to reduce these toxics [4]. For instance, between 2011 and 2021, European countries such as Austria, France, Germany, Latvia, and Spain reported an increase in pesticide sales [5], revealing difficulty in controlling plant pest attacks. Some of the most dangerous PPNs affect plant root systems, such as root-knot nematodes (*Meloidogyne*), cyst nematodes (*Globodera* and *Heterodera*), and root-lesion nematodes (*Pratylenchus*); therefore, nematicides are frequently applied to soil in the form of fumigant or non-fumigant formulations. Their efficacy is frequently suboptimal since treatments barely penetrate the topsoil, and the soil’s chemical properties heavily influence compound bioavailability and, consequently, their nematotoxicity [6]. Moreover, traditional nematicides have been alarmingly associated with a loss of soil biodiversity and concerns of acute toxicity in animals [7,8].

The first nematicides developed were mostly broad-spectrum fumigants that, although highly efficient, lacked specificity and compromised long-term agricultural sustainability [9]. For example, methyl bromide affected nematodes but also fungal pathogens, weeds, and soil arthropods, and it is now globally rejected. Some other old-generation fumigants are still in use, such as metam, which decomposes into the highly toxic volatile methyl isothiocyanate [10]; non-fumigant carbamates, e.g., oxamyl or aldicarb; and organophosphates, such as fosthiazate [11]. These non-fumigant pesticides are harmful to animals since they target acetylcholinesterase (AChE), a key enzyme in the animal cholinergic system [12]. Other currently used nematicides are obtained from the de novo design of natural compounds, e.g., avermectins, which derive from a fermentation process by the soil-dwelling actinomycete, *Streptomyces avermitilis* [13]. The hemisynthetic emamectin, from the avermectin family of compounds, is successfully employed against the pinewood nematode *Bursaphelenchus xylophilus*, a forest pathogen [14]. More recently, a next generation of synthetic nematicides, including fluazaindolizine, fluensulfone, or fluopyram, has been introduced that shows safer (eco)toxicological parameters [15]; however, their modes of action are still scarcely understood [1], which makes pinpointing the risks to agroecosystems a difficult task. Currently, the known molecular modes of action of nematicides are limited to AChE inhibition (e.g., oxamyl), glutamate-gated chloride channel allosteric modulation (e.g., emamectin benzoate), mitochondrial complex II electron transport inhibition (e.g., fluopyram), and acetyl CoA carboxylase inhibition (e.g., spirotetramat, an azaspiro compound) [16].

The many drawbacks of traditional pesticides highlight a need for novel nematicidal compounds that effectively tackle PPN diseases while safeguarding non-target organisms, e.g., beneficial plant growth-promoting microorganisms. The scarcity of commercially available alternatives might be countered by exploring the bionematicidal potential of natural products. Plants biosynthesise a large array of metabolites, either constitutively or induced by environmental stimuli, which serve as signals for communication or defence and are responsible for the ecological interplay between plants, insects, and microbes [17,18]. Phytochemicals are still a largely untapped source of nematicidal compounds [19] despite showing a high chemical diversity and specialised bioactivity [20,21]. For example, essential oils (EOs) are complex mixtures of plant volatiles with outstanding nematicidal potential. They are composed of plant natural products from diverse biosynthetic pathways, namely mono-, sesqui-, and a few diterpenes [22,23], and volatile phenylpropanoids, which are biosynthesised through the shikimate pathway [24]. But other types of chemical classes can occur in high amounts, such as aliphatic volatiles derived from fatty acids or β-keto acids, e.g., methyl ketones [25,26], or volatile sulphides, which are organosulfur compounds frequently found in *Allium* spp. (Amaryllidaceae) [27]. Currently, plant volatiles are largely used in the food, fragrance, and pharmaceutical industries [28]; however, despite their promising biopesticidal potential, their integration into plant protection products (PPPs) is still in its initial steps [29]. For instance, the monoterpenes geraniol and thymol were only recently introduced as bionematicides against root-knot nematodes, and sulphide-rich garlic extract was also adopted against PPN [30]. Nevertheless, a comprehensive understanding of the benefits that volatile phytochemicals might provide to the environment and animal life in comparison to traditional nematicides can contribute to the transition to sustainable nematicides.

To assess potential environmental risks, the present study compares a selection of highly nematicidal plant volatiles with widely used traditional synthetic nematicides by reviewing reported toxicological and ecotoxicological data and resorting to an in silico modelling approach based on reported experimental chemical properties. The reported environmental and human health safety thresholds provide insights into the benefits of novel bionematicides in contrast to the currently employed synthetic nematicides. Furthermore, in silico approaches aid in the prediction of the environmental and toxicological behaviours of these substances. By resorting to environmental modelling techniques, the predicted distribution of a toxicant in the environment can be gauged and understood [31]. Moreover, crossing exposure with reported or computed ecotoxicological data can provide a comprehensive view of the expected environmental benefits of biopesticides.

Ultimately, this comparative evaluation of plant volatiles against synthetic nematicides aims to highlight their potential for engineering safer and more effective bionematicides that can contribute to more sustainable agriculture practices, restore terrestrial ecosystems, and limit the loss of biodiversity.

## 2. Materials and Methods

### 2.1. Nematicidal Compounds

The comparative study reviewed and analysed experimental data reported by reputed online databases devoted to (eco)toxicology and resorted to specialised software for the in silico prediction of important parameters that allow for the environmental risks and potential human health benefits of twenty nematicidal plant volatiles (from four chemical or biosynthetic classes) to be gauged in comparison to five traditional nematicides.

#### 2.1.1. Volatiles with High Nematicidal Activity

**Figure 1:**
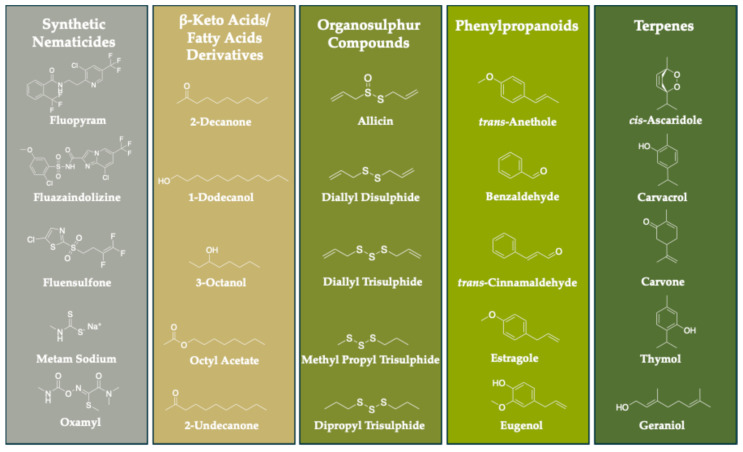
The chemical structures of the phytochemical volatiles and traditional nematicides.

Five compounds reported as strong nematicides were selected for each of the terpene, phenylpropanoid, organosulphur compound, and β-keto acid/fatty acid derivative groups (Figure 1).

#### 2.1.2. Traditional Nematicidal Pesticides

The phytochemicals were compared to five nematicides, which were selected to represent old- and next-generation pesticides (Figure 1). The carbamate oxamyl is an old-generation systemic pesticide that is generally applied to crop soil as granules; metam sodium is an old-generation fumigant pesticide used as a nematicide, herbicide, and fungicide. The fluorinated pesticides fluazaindolizine, fluensulfone, and fluopyram were chosen as the three principal and most promising next-generation nematicides [1].

### 2.2. Assessment of Environmental Impacts

The prediction of the environmental safety of a chemical substance for widespread use is very complex since natural conditions present a high number of variables that can influence its toxicity towards non-target organisms and its environmental dispersion. However, several databases host experimental data on the acute toxicity thresholds for model species of the most important organism groups in many ecosystems, and they provide a good assessment of their impacts on natural populations. Additionally, in silico models can predict important environmental parameters regarding the distribution and fate of a substance based on its chemical properties, many of which are available in online databases.

#### 2.2.1. Reported Experimental Thresholds for Ecotoxicity in Model Organisms

The determination of acute toxicity to aquatic organisms is required for gauging environmental hazards and to conduct a risk assessment of plant protection products, which is mandatory for EU legislation. Toxicity thresholds are generally obtained on model organisms for three important trophic levels: primary producers, such as algae or plant species; primary consumers/secondary producers, using invertebrates such as *Daphnia* spp.; and secondary consumers, by screen toxicity on vertebrates, namely species of fish. For EU chemical legislation, testing acute aquatic toxicity is a basic requirement, and it is usually performed through short-term exposure of the organism to a series of concentrations of a target substance that yields an EC_50_ value. These parameters are freely hosted by reputed databases, such as the European Chemicals Agency (ECHA) database [32], the PPDB (the Pesticide Properties Database) [33,34], and PubChem [35], which were used for the present work.

In the case of a lack of data in the repository, the Toxicity Estimation Software Tool (T.E.S.T.) version 5.1.2. (Washington, DC, USA), which is freely available at the US EPA website [36], was used to predict the LD_50_ value of the chemical against the fathead minnow for 96 h, an organism model for fish, and the LC_50_ value against *Daphnia magna* for 48 h (a model for invertebrates).

#### 2.2.2. Predicted Environmental Distribution

The chemical characteristics of the target compounds were used to assess their potential dispersion through the most important environmental compartments. Predicted environmental distribution (PED) percentages of the nematicidal volatiles were determined through the equilibrium criterion model suggested by Mackay et al. [31] and compared to that of the selected synthetic nematicides. Supported by this model, PED percentages were computed using the Level I Mackay Fugacity Model beta version 4.39, Trent University, Canada [37]. This model was set to predict a situation in which a fixed quantity of a compound, theoretically 100,000 kg, is introduced to a closed system under steady-state and equilibrium conditions at 25 °C. The physical and chemical properties as well as the partition coefficients required for each compound to compute their respective PED values were, namely, the molecular mass (g/mol), melting point (°C), vapour pressure (Pa), solubility in water (mg/L), air/water partition coefficient or Henry’s law constant (Pa.m^3^/mol), *n*-octanol/water partition coefficient (log value of Kow), and soil organic carbon/water partition coefficient (Koc). These values were retrieved from the PubChem online database [33] and the PPDB, or the Pesticide Properties Database [34] (Table 1). When needed, a predictive approach was used for the calculation of these parameters by resorting to the EPISuite™ software (v. 4.11) [38], which is freely available from the US Environmental Protection Agency.

### 2.3. Potential Benefits to Human Health

The risks of phytochemicals to human health were compared to those of synthetic nematicides by reviewing the reported toxicological parameters, namely the acute oral and dermal toxicity thresholds, which provide valuable information on appropriate safety measures and regulatory requirements for handling and use and the in silico prediction of the toxicity profiles of xenobiotics.

#### 2.3.1. Reported Experimental Acute Toxicity Thresholds

Acute toxicity is experimentally determined by resorting to animal studies, and the results are used to determine the potential risks to human health. Acute toxicity can be measured through various routes of exposure, with the oral and dermal toxicity routes being the most used. Acute oral toxicity is commonly expressed as the dose of a compound that is administered through the mouth and is lethal to 50% of the tested animals within a specified time period (LD_50_). Results are employed to determine the appropriate labelling and safety precautions for consumer products and industrial chemicals. Acute dermal toxicity determines the median lethal dose (LD_50_) that causes toxic effects through skin exposure. Several databases host experimental data on these parameters. For the present work, data were retrieved from reputed databases, namely the European Chemicals Agency (ECHA) database [32], the PPDB (the Pesticide Properties Database) [33,34], and PubChem [35].

#### 2.3.2. Computed Toxicological Traits

Undesirable toxicity traits can easily be predicted in the initial phases of drug design and development to provide insights into the probable active compound suitability. By resorting to specialised software, the harmful effects on human health can be gauged. Also, computational toxicity estimations are not only faster than the determination of toxic doses in animals but can also help to reduce the amount of animal experiments.

The prediction of toxicological parameters, namely the toxicity class, hepatotoxicity, neurotoxicity, respiratory toxicity, cardiotoxicity, mutagenicity, and the developmental toxicity of the compounds, was performed through an in silico approach by resorting to specialised certified software developed for the determination of the toxicity parameters of xenobiotics.

The webtool ProTox 3.0 is based on the SuperToxic database, which provides a comprehensive collection of toxins, namely ca. 60,000 structures with corresponding properties from different sources (animals, plants, synthetic, etc.), combined with chemical features that enable the detailed investigation of correlations between the chemical, functional, and structural properties of compounds [40,41]. Toxicity classes are defined according to the globally harmonised system of classification of labelling of chemicals (GHS). Compounds that are considered fatal if swallowed are added to class 1 (LD_50_ ≤ 5 mg/kg) or 2 (5 < LD_50_ ≤ 50 mg/kg), compounds considered toxic if swallowed belong to class 3 (50 < LD_50_ ≤ 300 mg/kg), compounds considered harmful if swallowed belong to class 4 (300 < LD_50_ ≤ 2000 mg/kg) or 5 (2000 < LD_50_ ≤ 5000 mg/kg), and those that are non-toxic belong to class 6 (LD_50_ > 5000 mg/kg). The model for hepatotoxicity uses the drug-induced liver injury prediction type with the Random Forest machine learning algorithm with the SMOTE TC (Synthetic Minority Over-Sampling using Tanimoto Coefficient) sampling method. The model for neurotoxicity uses the chemical-induced neurotoxicity prediction type with Random Forest and the SMOTEVDM sampling method. The model for cardiotoxicity uses the chemical-induced cardiotoxicity prediction type with Random Forest and the SMOTE TC sampling method. The model for respiratory toxicity uses the chemical-induced respiratory toxicity prediction type with Random Forest and the randOS sampling method [40,41]

The Toxicity Estimation Software Tool (T.E.S.T.) version 5.1.2. (Washington, DC, USA) is freely available on the US EPA website [36]. Predictions are based on quantitative structure–activity relationship (QSAR) models by crossing an extensive review of data on the Ames mutagenicity test, which is based on the *Salmonella typhimurium* reverse mutation assay [42], and on developmental toxicity, which is based on the Food and Drug Administration’s (FDA) classification of a risk factor [43], with known molecular descriptors of physical and chemical characteristics of the compounds. The software assessed mutagenicity and developmental toxicity through the hierarchical cluster method in which the toxicity of a given compound is estimated using the weighted average of the predictions from several different models, which were obtained by using Ward’s method to divide the training set into a series of structurally similar clusters [43]. A genetic algorithm-based technique is used to generate models for each cluster. This software was also used to predict the values of acute oral toxicity when no information was found in the selected repositories.

## 3. Results

### 3.1. Reported Nematicidal Activities

The activity of compounds against PPNs is generally screened through direct contact bioassays in which nematode life stages are put into contact with the chemical at different concentrations for an amount of time, and mortality is assessed. Ultimately, a half maximal effective concentration (EC_50_) value can be determined that is comparable to that of other compounds reported in other works. In the present work, the most active volatile phytochemicals were reviewed for four important classes of compounds, namely terpenes, phenylpropanoids, organosulfur compounds, and β-keto acid/fatty acid derivatives, in comparison to five traditional old- and next-generation synthetic nematicides. Overall, the pesticides were reported to possess higher activity against important PPNs than most of the nematicidal phytochemicals. The highest activities reported were for fluopyram, a next-generation nematicide, against *M. incognita* and *M. enterolobii* (Table 2). Similarly, high activities were reported for fluensulfone, oxamyl, and metam sodium against *M. incognita* with EC_50_ values in the range of 0.001 to 0.008 mg/mL. Among the volatile phytochemicals, comparable activities were only reached by diallyl and dipropyl trisulphide with EC_50_ values ranging from 0.003 to 0.005 mg/mL, and the fatty acid derivative 1-dodecanol with an EC_50_ value of 0.009 mg/mL, against the pinewood nematode, and the phenylpropanoid benzaldehyde with an EC_50_ value of 0.009 mg/mL against *M. incognita*.

### 3.2. Environmental Safety

To assess the ecological risks of the phytochemicals or traditional nematicides to non-target organisms, data on the potential environmental fate of these compounds (Table 3) were crossed with the reported experimental toxicity thresholds for the model organisms of the main trophic levels (Table 4).

The predicted environmental distribution of traditional nematicides tends to differ from the majority of the analysed phytochemical volatiles. Fluazaindolizine, fluensulfone, metam sodium, and oxamyl show a prevalent affinity towards the water environmental compartment, while fluopyram was predicted to disperse to both the soil (59.3%) and water (39.3%) environmental compartments. Unlike traditional nematicides, the volatile terpenes were predicted to have a higher affinity to the air (*cis*-ascaridole, carvone, and geraniol) or the soil (carvacrol and thymol) environmental compartments.

Phenylpropanoids also showed lower affinity to the water compartment than the traditional nematicides, with some also dispersing to the air (*trans*-anethole) and soil (estragole) environmental compartments. Among the organosulfur compounds, only allicin was predicted to show a very high affinity to the water environmental compartment, and the sulphides either showed a higher affinity to the air (diallyl disulphide, diallyl trisulphide, and methyl propyl trisulphide) or the soil (dipropyl trisulphide) environmental compartments. In the case of the β-keto acid or fatty acid derivatives, 2-decanone and octyl acetate showed a higher affinity to the air environmental compartment, 1-dodecanol and 2-undecanone showed a higher affinity to the soil environmental compartment, and 3-octanol showed a higher affinity to the water environmental compartment.

Biodiversity in groundwater and aquifers is generally heavily affected by pesticide overuse given their high affinity to water (Table 3). Evaluating the acute effects on aquatic non-target organisms is a fundamental step in understanding the potential for environmental hazards. The acute ecotoxicological thresholds reported for aquatic trophic groups were reviewed for the volatile phytochemicals in comparison to traditional nematicides (Table 4). Overall, from the synthetic nematicides, oxamyl, metam sodium, and fluopyram were reported to be more toxic than fluazaindolizine or fluensulfone. For fish, the reported LC_50_ values were ca. 10-, 150-, and 30-fold lower for oxamyl, metam sodium, and fluopyram when compared to fluazaindolizine or fluensulfone (Table 4). The toxicity thresholds reported for algae were very low for most pesticides, with the exception of fluazaindolizine (46 mg/L in comparison to 0.02–1.1 mg/L). For *Daphnia magna* (a model organism for invertebrates), the reported LC_50_ values were ca. 400-, 120-, and 150-fold or ca. 180-, 50-, and 70-fold lower for oxamyl, metam sodium, and fluensulfone when compared to fluazaindolizine or fluopyram, respectively.

Overall, the analysed volatiles showed higher toxicity thresholds than most of the toxic pesticides, but only carvone was less lethal than all of the selected pesticides across the model organisms. For fish, terpene carvone stood out for its higher LC_50_ value (50 mg/L in comparison with >30 or 38 mg/L for fluazaindolizine or fluensulfone, respectively) (Table 4). For algae, only carvone (154.7 mg/L) and 3-octanol (114.4 mg/L), a fatty acid derivative, were reported to have higher thresholds of toxicity than the pesticides reviewed. Against *Daphnia magna*, the model organism for invertebrates, most compounds were reported to have low toxicity thresholds with the exception of the pesticides fluazaindolizine (>120 mg/L) and fluopyram (55.5 mg/L); the fatty acid derivative 3-octanol (184.5 mg/L); allicin (38.7 mg/L), an organosulfur compound; and the terpenes carvone (249.5 mg/L) and *cis*-ascaridole (40.3 mg/L).

### 3.3. Benefits to Human Health

Among the reported oral acute toxicities, the groups that pose the greatest risks were are selected pesticides and organosulfur compounds, particularly the sulphides. While the synthetic pesticide oxamyl stands out for its very high risk, fluopyram shows a lower oral acute toxicity LD_50_ value (Table 5). The next-generation nematicides fluazaindolizine, fluensulfone, and fluopyram tended to show safer characteristics against mammals than the old-generation compounds. Overall, the selected terpenes, phenylpropanoids, and derivatives of β-keto acids or fatty acids had higher reported LD_50_ values, suggesting that they have a lower risk in the case of oral exposure. The data reported for dermal toxicity was limited, with the group of organosulfur compounds being less reported. The analysed compounds showed relatively high thresholds for dermal toxicity and can be considered equally impactful. In fact, through a globally harmonised system (GHS) of classification and the labelling of hazardous substances, compounds can be assigned to one of five hazard categories based on acute toxicity by the oral, dermal, or inhalation route according to numeric cut-off criteria [59]. For acute oral toxicity, compounds with LD_50_ values below 5 mg/kg are classified as category 1 toxicants, those with LD_50_ values between 5 and 50 mg/kg are classified as category 2, those with LD_50_ values between 50 and 3000 mg/kg are classified as category 3, those with LD_50_ values between 300 and 2000 mg/kg are classified as category 4, and those with LD_50_ values above 2000 mg/kg are classified as category 5. For the analysed compounds, with the exception of oxamyl (a category 1 toxicant), they can all be classified as category 3 or above toxicants. For acute dermal toxicity, compounds with LD_50_ values below 50 mg/kg are classified as category 1 toxicants, those with LD_50_ values between 50 and 200 mg/kg are classified as category 2, those with LD_50_ values between 200 and 1000 mg/kg are classified as category 3, those with LD_50_ values between 1000 and 2000 mg/kg are classified as category 4, and those with LD_50_ values above 2000 mg/kg are classified as category 5. For the analysed compounds and with the available information, no substance was classified below category 5.

The predictive approach based on computational toxicology revealed that terpenes and phenylpropanoids are expected to induce a higher number of negative impacts on organ functioning than other compounds, even though their toxicity class suggests that they are safer than the pesticides. Overall, the β-keto acid/fatty acid derivatives were the compounds with the least toxicological impacts, indicating a potential for the development of safer biopesticides (Table 6). The prediction of mutagenicity and developmental toxicity revealed that among the analysed compounds, only the pesticide oxamyl showed a risk of inducing mutation, while all compounds showed a potential to induce developmental toxicity with the exceptions of the terpene geraniol, the phenylpropanoid benzaldehyde, the sulphide diallyl disulphide, and the aliphatic alcohol 1-dodecanol (Table 6).

## 4. Discussion

Most old-generation pesticides were not initially discovered as nematicides, but rather as sterilants or fumigants, insecticides, fungicides, or animal health drugs, so the history of nematicides has been one of accidental discoveries. This has resulted in an underdeveloped industry, which has only recently turned its attention to the design of precision nematicidal compounds. A new focus on nematicide development is, in large part, a response to the overall increasing regulatory pressure on hazardous products (traditional class 1 nematicides) and, more specifically, the fact that some of the most effective and popular nematicides, including methyl bromide, fenamiphos, and aldicarb, are now severely restricted [11]. Nematicides have become a global priority, and many industries are now allocating significant research funding to discover novel active substances. These must be very different from previous products as they must be more selective, they must only target nematodes, be less toxic, and be safer to use. The present study focused on gauging the environmental benefits and risks of using plant volatiles as nematicidal agents.

Plant volatiles and traditional nematicides present differential chemical features that influence their bioactivity, environmental distribution, and (eco)toxicological hazards. While both natural and synthetic compounds can be applied to tackle PPN, their toxicological profiles tend to be dissimilar. Among the traditional pesticides, oxamyl was seen to be the most dangerous compound. Even though this class 1 pesticide has high nematicidal activity against soil nematodes, it has a strong affinity to the water compartment and very high toxicity to non-target organism groups, namely fish, algae, and invertebrates. Against mammals, the oral toxicity threshold was much lower than the other analysed pesticides, and although it was not predicted to induce toxicity to organ functioning, it appears to potentially induce mutagenicity and developmental problems. In fact, among the three commonly used agricultural pesticides (2,4-dichlorophenoxy acetic acid (2,4-D), dicamba, and oxamyl), only oxamyl was reported to induce genomic DNA damage in a concentration-dependent manner [60]. However, the soil composition was reported to impact oxamyl persistence and toxicity [61], which can lead to inconclusive assessments of potential risks, especially regarding the persistence and toxicity of its degradation residues, e.g., oxamyl oxyme or dimethyl amino oxoacetic acid, which are already quantified in concerning amounts in drinking water [62]. Thus, oxamyl might be responsible for long-term environmental effects since it was additionally predicted to have a strong persistence in the environment in comparison to volatile phytochemicals [45]. Fluopyram is a next-generation nematicide that appears to have very strong activity against soil parasitic nematodes without many of the harmful effects of oxamyl. Besides having a lower affinity to the water environmental compartment, its toxicity to invertebrates was also reported to be lower. The oral toxicity threshold for mammals was high and was only predicted to exert developmental toxicity. However, fluopyram degradation is believed to be slow, as it has been found in soil depths below 30 cm and can leach and contaminate groundwater depending on the soil composition [63]. Additionally, not much is known about the toxicity of its degradation metabolites that can contribute to its environmental impacts [64]. Fluazaindolizine, another next-generation pesticide, shows higher environmental safety compared to the remaining pesticides since its reported toxicity thresholds to non-target aquatic model organisms are higher; however, its nematicidal activity is reported to be lower. For example, in a field test where fluazaindolizine, fluensulfone, fluopyram, oxamyl, and metam potassium were compared for the control of root-knot nematodes in cucumber and squash, only metam seemed to lower nematode infection and increase yield, and the water solubility of these compounds most probably exerted some influence [65]. Although fluazaindolizine seems to have some advantages over other pesticides, the techniques used for quantifying its degradation metabolites are only now being optimised, and information on their toxicological impacts is still being analysed.

The phytochemical volatiles with the highest activity were organosulfur compounds, mainly sulphides. Sulphides were predicted to disperse mostly to the air environmental compartment, suggesting a predisposition to be used as fumigants and low environmental persistence. Although their reported toxicity thresholds to aquatic non-target organisms are low, the lower predicted dispersion into the water environmental compartment makes their use a possible non-risk to aquatic life in the long term. Against mammals, sulphides are reported to have considerable toxic effects through the oral route of exposure. Although diallyl disulphide and diallyl trisulphide were predicted to have hepatotoxicity, experimental studies have reported that they have a protective effect against strongly hepatotoxic substances in mammals [66,67]. Nematicidal formulations are currently commercialised based on garlic extracts due to their richness in allicin and polysulphides. However, their efficacy appears to be relatively dependent on environmental conditions, particularly on edaphic parameters [68].

The analysed terpenes and phenylpropanoids are common components of essential oils. They have considerable biological activities against PPN and relatively high toxicity thresholds to aquatic organisms, with carvone and *cis*-ascaridole showing the best values. Despite having high reported oral and dermal toxicity thresholds, they were predicted to negatively influence organ functioning, with positive results for hepatotoxicity, neurotoxicity, cardiotoxicity and respiratory toxicity, which suggests that, as bionematicides, the formulations of these compounds should be handled with care by farmers. A bionematicide is commercially available based on the activities of the monoterpenes thymol and geraniol. Although its mode of action is still not understood, terpene activity is not only considerable for second-stage juveniles but also for the inhibition of hatching [69]. In previous works, the acyclic monoterpenic primary alcohol geraniol showed higher activities than tertiary alcohols against J_2_ root-knot nematodes, such as *M. incognita*, *M. javanica*, and *M. ethiopica* [70,71,72]. The monoterpene aldehydes citral and citronellal were also seen to exert strong nematicidal activities against *M. ethiopica* while possessing a lower predicted affinity to the water environmental compartment compared to the synthetic nematicides oxamyl, metam sodium, or fluopyram, and they possess higher toxicity thresholds against aquatic model organisms, which may indicate a lower persistence in the interstitial water in the soil or bodies of water [73]. As components of essential oils, the monoterpene carvacrol and the phenylpropanoid *trans*-anethole are suggested to function synergistically against the pinewood nematode [74]. Besides showing higher nematicidal activities than the commercial nematicide emamectin benzoate, they were reported to have higher toxicity thresholds to mammals and were predicted to disperse to the air, soil, and water environmental compartments. Studying terpene synergistic interactions has good potential to contribute to the development of precision biopesticides with optimised nematicidal activity and lower environmental risks.

The derivatives of fatty acids or β-keto acids were seen to hold the best properties for the development of biopesticides. They can have strong nematicidal activities (1-dodecanol), lower dispersion in the water environmental compartment alongside lower toxicity to aquatic organisms (3-octanol), and show relatively safe toxicity thresholds for mammals or predicted interference with important organic functions. In previous works with the root-lesion nematode *Pratylenchus penetrans*, the fatty alcohol 3-octanol was responsible for high nematicidal activities, which is notable given that root-lesion nematodes appear to have strong resistance to volatiles as nematicidal compounds of direct contact activity [45]. The nematicidal volatiles analysed in this study, which included fatty alcohols such as 1-decanol, 1-undecanol, or 1-dodecanol, were found to have higher nematicidal activities than oxamyl, higher affinity to the air and soil environmental comparts, and overall safer (eco)toxicological parameters.

In the present study, several volatiles were predicted to show enhanced affinity to the soil environmental compartment. Thus, assessing the detrimental impacts on soil-dwelling non-target organisms is also a good method to evaluate environmental safety. This evaluation is commonly performed by screening activity against earthworms (*Eisenia fetida*) or insects. For example, the essential oils of *Oliveria decumbens*, *Thymus daenensis*, *Satureja sahendica*, *S. khuzistanica*, and *S. rechingeri*, which are rich in the monoterpenes thymol and carvacrol, were evaluated against three insects of economic relevance, and their toxicity was also assessed towards the earthworm *Eisenia fetida* [75]. The essential oils showed good insecticidal activities, and their toxicity to earthworms, at a concentration of 200 mg/kg of soil for 14 days, was reported to be weak or absent, while the synthetic pyrethroid insecticide α-cypermethrin induced complete mortality at a concentration of 0.1 mg/kg of soil on the 14th day, which indicates their potential use for the development of safer insecticidal formulations. In another study, the insecticidal activity of *Stevia rebaudiana* essential oil, which is rich in the sesquiterpenes caryophyllene oxide, spathulenol, and nerolidol, was assessed alongside its toxicity towards *E. fetida*. The essential oil, at a concentration of 200 mg/kg for 14 days, was unable to induce any noticeable effects on *E. fetida*. Likewise, its toxicity against the non-target harlequin ladybug *Harmonia axyridis* (Coleoptera: Coccinellidae) was analysed and determined to induce no measurable toxicity at a concentration of 10.8 mL/L, whereas α-cypermethrin induced a mean mortality of 77.5% at just 6.4 × 10^−6^ mL/L [76].

In summary, an integrated approach was proposed based on the reported ecotoxicological parameters and predictive software to screen for nematicidal volatile phytochemicals as bionematicides. This is the first report, to the best of our knowledge, in which these approaches are applied for the comparison of phytochemical volatiles and commercial nematicides on their environmental off-target effects. Ultimately, a judicious filtering of bionematicidal compounds at an initial phase of development might contribute to more efficiently direct research efforts as well as hasten the approval and implementation of plant volatiles as eco-friendly PPPs.

## 5. Conclusions

Plant volatiles are still mostly unexploited natural resources to tackle PPN diseases. While in vitro bioactivity is well established for several compounds, most of their putative detrimental impacts have yet to be thoroughly analysed. The comparative evaluation between bionematicidal volatiles and synthetic nematicides underlined their favourable environmental characteristics, including their low risk to mammals and favourable environmental fate based on in silico modelling. Fatty acid derivatives stood out for their high nematicidal activity, low predicted environmental impacts, and low toxicity to non-target organisms, while some compounds of the terpene, phenylpropanoid, or organosulphur chemical classes still showed potential for toxicity to aquatic organisms. Overall, using plant volatiles can constitute a powerful means to control PPN in a sustainable way, safeguarding soil health and biodiversity.

## Figures and Tables

**Table 1:** The physicochemical properties and partition coefficients of the nematicidal plant volatiles and traditional pesticides required to perform the Level I Mackay Fugacity Model [37] [molecular mass (g/mol), melting point (°C), vapour pressure (Pa), solubility in water (mg/L), air/water partition coefficient or Henry’s Law constant (Pa.m^3^/mol), *n*-octanol/water partition coefficient (logKow), and soil organic carbon/water partition coefficient (Koc)].

	CAS Number	Molecular Mass (g/mol)	Melting Point (°C)	Vapour Pressure (Pa)	Solubility in Water (mg/L)	Henry’s Law Constant (Pa.m^3^/mol)	logK_OW_ (Unitless)	K_OC_^1^ (Unitless)
*Pesticides*								
Oxamyl	23135-22-0	219.26	98.5	1.8 × 10^−5^	148,100	2.66 × 10^−8^	−0.44	0.3
Metam sodium	137-42-8	129.19	88.5	0.06	578,290	1.30 × 10^−5^	−2.91	4 × 10^−4^
Fluazaindolizine	1254304-22-7	468.20	218.5	0.21	2148	0.046	–0.16	0.2
Fluensulfone	318290-98-1	291.70	34.4	0.03	545	0.016	1.96	2
Fluopyram	658066-35-4	396.76	117.5	1.2 × 10^−6^	16	2.98 × 10^−5^	3.30	698
*β-Keto acid/fatty acid derivatives*								
2-Decanone	693-54-9	156.26	14.0	0.33	77	0.672	3.70	1750
1-Dodecanol	6175-49-1	184.32	21.0	0.11	4	5.207	5.13	47,200
3-Octanol	589-98-0	130.23	−45.0	0.34	1380	3.209	2.72	184
Octyl acetate	112-14-1	172.26	−38.5	0.53	179	0.513	3.40	879
2-Undecanone	112-12-9	170.29	15.0	5.34	80	0.114	3.69	1710
*Organosulfur compounds*								
Allicin	539-86-6	162.30	25.0	1.80 ^2^	24,000	0.012	1.30	7
Diallyl disulphide	2179-57-9	146.30	−24.4 ^2^	0.01	71 ^2^	0.027	2.20	56
Diallyl trisulphide	2050-87-5	178.30	8.4 ^2^	0.14	51 ^2^	0.499	2.60	139
Dipropyl trisulphide	6028-61-1	182.40	11.0 ^2^	3.20	28	0.209	3.84 ^2^	2420
Methyl propyl trisulphide	17619-36-2	154.32	−11.0 ^2^	0.22	265 ^2^	0.130	2.20	56
*Phenylpropanoids*								
*trans*-Anethole	4180-23-8	148.20	21.0	6.67	111	8.905	3.33	748
Benzaldehyde	100-52-7	106.12	−57.1	0.01	6950	2.031	1.48	106
*trans*-Cinnamaldehyde	104-55-2	132.16	−18.0	3.90	2865	0.180	2.11	45
Estragole	140-67-0	148.20	−1.2	6.67	178	5.553	3.47	1030
Eugenol	97-53-0	164.20	−9.2	2.95	2460	0.196	2.49	108
*Terpenes*								
*cis*-Ascaridole	512-85-6	168.23	3.3	0.02	530	0.760	2.30	698
Carvacrol	499-75-2	150.22	1.0	3.09	1250	0.370	3.33	748
Carvone	99-49-0	150.21	−43.0	1.90	27	0.105	2.40	88
Thymol	89-83-8	150.22	49.6	2.10	980	0.322	3.30	698
Geraniol	106-24-1	154.25	−15.0	4.00	100	6.170	2.90	278

^1^ Estimated using the Seth method [39]. ^2^ Computed using the US Environmental Protection Agency’s EPISuite™ software (v. 4.11) [38].

**Table 2:** The reported nematicidal activities (half maximal effective concentrations, EC_50_) of the phytochemical volatiles against important plant-parasitic nematodes (PPNs), namely *Bursaphelenchus xylophilus*, *Meloidogyne incognita*, *M. javanica*, *M. enterolobii*, and *Pratylenchus penetrans*.

Compounds	PPNs	EC_50_ (mg/mL)	Reference
*Pesticides*			
Oxamyl	*M. enterolobii*	0.028	[44]
	*M. incognita*	0.004	[44]
	*P. penetrans*	66 ± 2 ^1^	[45]
Metam sodium ^2^	*M. incognita*	0.008	[46]
Fluazaindolizine	*M. enterolobii*	>0.200	[44]
	*M. incognita*	0.030	[44]
Fluensulfone	*M. enterolobii*	0.026	[44]
	*M. incognita*	0.001	[44]
Fluopyram	*M. enterolobii*	0.0004	[44]
	*M. incognita*	0.0001	[44]
*β-Keto acid/fatty acid derivatives*			
2-Decanone	*M. incognita*	0.056	[47]
	*M. javanica*	0.056	[47]
1-Dodecanol	*B. xylophilus*	0.009	[48]
3-Octanol	*P. penetrans*	0.680	[45]
Octyl acetate	*M. incognita*	0.037	[47]
	*M. javanica*	0.061	[47]
2-Undecanone	*M. incognita*	0.021	[47]
	*M. javanica*	0.023	[47]
*Organosulfur compounds*			
Allicin	*M. incognita*	0.018	[49]
Diallyl disulphide	*B. xylophilus*	0.043–0.047	[50]
Diallyl trisulphide	*B. xylophilus*	0.003–0.004	[50]
Dipropyl trisulphide	*B. xylophilus*	0.004–0.005	[51]
Methyl propyl trisulphide	*B. xylophilus*	0.017–0.023	[51]
*Phenylpropanoids*			
*trans*-Anethole	*M. incognita*	0.170	[52]
	*P. penetrans*	1.780	[45]
Benzaldehyde	*M. incognita*	0.009	[53]
	*P. penetrans*	0.450	[45]
*trans*-Cinnamaldehyde	*B. xylophilus*	0.057	[54]
	*M. incognita*	0.064	[55]
Estragole	*M. incognita*	0.230	[53]
Eugenol	*M. incognita*	0.256	[52]
	*P. penetrans*	1.720	[45]
*Terpenes*			
*cis*-Ascaridole	*M. incognita*	0.033	[56]
Carvacrol	*B. xylophilus*	0.097–0.125	[57]
	*M. incognita*	0.112	[52]
	*P. penetrans*	0.480	[45]
Carvone	*M. incognita*	0.115	[52]
Thymol	*B. xylophilus*	0.110–0.119	[57]
	*M. javanica*	0.140	[58]
	*P. penetrans*	0.500	[45]
Geraniol	*B. xylophilus*	0.415–0.540	[57]
	*M. incognita*	0.158	[52]

^1^ Mean corrected mortality (%) reported at 2 mg/mL; ^2^ values were reported for its decomposition volatile metabolite, methyl isothiocyanate.

**Table 3:** The predicted environmental distribution (PED, %) computed through level I of Mackay’s fugacity model [31].

	Predicted Environmental Distribution (%)						
	Aerosols	Air	Biota	Sediment	Soil	Suspended Particles	Water
*Pesticides*							
Oxamyl	0.0	0.0	0.0	0.0	0.0	0.0	99.9
Metam Sodium	0.0	0.0	0.0	0.0	0.0	0.0	99.9
Fluazaindolizine	0.0	0.9	0.0	0.0	0.0	0.0	99.0
Fluensulfone	0.0	0.3	0.0	0.1	6.4	0.0	93.1
Fluopyram	0.0	0.0	0.0	1.3	59.3	0.0	39.3
*β-Keto acid/fatty acid derivatives*							
2-Decanone	0.0	73.5	0.0	0.5	20.6	0.0	5.4
1-Dodecanol	0.0	1.0	0.0	2.1	95.9	0.0	0.9
3-Octanol	0.0	31.5	0.0	0.4	19.3	0.0	48.7
Octyl acetate	0.0	77.9	0.0	0.3	14.3	0.0	7.5
2-Undecanone	0.0	32.4	0.0	1.2	52.2	0.0	14.1
*Organosulfur compounds*							
Allicin	0.0	0.2	0.0	0.0	1.5	0.0	98.2
Diallyl disulphide	0.0	98.0	0.0	0.0	0.2	0.0	1.8
Diallyl trisulphide	0.0	88.3	0.0	0.0	2.7	0.0	8.9
Dipropyl trisulphide	0.0	39.8	0.0	1.1	49.6	0.0	9.5
Methyl propyl trisulphide	0.0	70.1	0.0	0.0	3.2	0.0	26.6
*Phenylpropanoids*							
*trans*-Anethole	0.0	40.4	0.0	0.8	36.3	0.0	22.5
Benzaldehyde	0.0	28.6	0.0	0.0	1.6	0.0	69.8
*trans*-Cinnamaldehyde	0.0	3.2	0.0	0.2	8.6	0.0	88.0
Estragole	0.0	25.4	0.0	1.1	50.7	0.0	22.7
Eugenol	0.0	3.1	0.0	0.4	18.3	0.0	78.2
*Terpenes*							
*cis*-Ascaridole	0.0	93.0	0.0	0.0	0.9	0.0	6.1
Carvacrol	0.0	2.7	0.0	1.3	59.2	0.0	36.7
Carvone	0.0	64.1	0.0	0.1	5.7	0.0	30.1
Thymol	0.0	2.5	0.0	1.3	57.8	0.0	38.3
Geraniol	0.0	43.5	0.0	0.5	21.0	0.0	35.0

**Table 4:** The reported acute ecotoxicological thresholds (median lethal/effective concentration, LC_50_/EC_50_, mg/L) for non-target aquatic model organisms (fish, algae, and invertebrates), retrieved from the ECHA online database [32], the PPDB (the Pesticide Properties Database) [33,34], and PubChem [35].

	Fish	Algae	Invertebrates ^9^
	LC_50_ (mg/L)	EC_50_ (mg/L)	EC_50_ (mg/L)
*Pesticides*			
Oxamyl	3.1 ^1^	0.9 ^6^	0.3
Metam sodium	>0.2 ^2^	1.1 ^6^	1.0
Fluazaindolizine	>30.0 ^3^	46.0 ^6^	>120
Fluensulfone	38.0 ^1^	0.02 ^6^	0.8
Fluopyram	>1.0 ^4^	>1.1 ^7^	55.5
*β-Keto acid/fatty acid derivatives*			
2-Decanone	5.0 ^4,^*	-	4.6 *
1-Dodecanol	1.0 ^4^	-	0.8
3-Octanol	23.2 ^4,^*	114.4 ^6^	184.5
Octyl acetate	8.2 ^4,^*	-	6.4 *
2-Undecanone	3.0 ^1^	>0.2 ^6^	0.2
*Organosulfur compounds*			
Allicin	23.9 ^4,^*	-	38.7 *
Diallyl disulphide	5.0 ^4,^*	-	2.3 *
Diallyl trisulphide	1.9 ^4,^*	-	0.4 *
Dipropyl trisulphide	1.8 ^4,^*	-	0.2 *
Methyl propyl trisulphide	6.8 ^4,^*	-	1.0 *
*Phenylpropanoids*			
*trans*-Anethole	7.0 ^5^	9.6 **	4.2
Benzaldehyde	1.1 ^2^	33.1 ^6^	19.7
*trans*-Cinnamaldehyde	>20.0 ^2^	16.1 ^8^	11.5
Estragole	5.3 ^4,^*	-	3.8 *
Eugenol	>10.0 ^1^	15.4 ^6^	1.1
*Terpenes*			
*cis*-Ascaridole	10.9 ^4,^*	-	40.3 *
Carvacrol	6.2 ^5,^**	4.1 ^6^	6.1
Carvone	50.0 ^5^	154.7 ^6^	249.5
Thymol	3.0 ^1^	11.7 ^6^	4.9
Geraniol	11.6 ^1^	48.0 ^6^	16.1

The values reported for the fish species ^1^ Oncorhynchus mykiss, ^2^ Lepomis macrochirus, ^3^ Cyprinodon variegatus, ^4^ Pimephales promelas, and ^5^ Danio rerio; the algae ^6^ Raphidocelis subcapitata, ^7^ Skeletonema costatum, and ^8^ Chlorella vulgaris; and the invertebrate ^9^ Daphnia magna. * The values were predicted using the T.E.S.T. software (v. 5.1.2.) [36] and the hierarchical clustering method. ** The model organism was not reported.

**Table 5:** Reported oral and dermal toxicity thresholds for mammals (median lethal dose, LD_50_, mg/kg) [32,33,34,35].

Compounds	Reported Oral Toxicity (mg/kg) ^1^	Reported Dermal Toxicity (mg/kg) ^2^
*Pesticides*		
Oxamyl	3	5000
Metam sodium	896	2000
Fluazaindolizine	940	2000
Fluensulfone	671	>2000
Fluopyram	>2000	2000
*β-Keto acid/fatty acid derivatives*		
2-Decanone	7936	-
1-Dodecanol	>12,800	>8000
3-Octanol	>5000	>5000
Octyl acetate	3000	>5000
2-Undecanone	5000	>5000
*Organosulfur compounds*		
Allicin	1121 ^3^	-
Diallyl disulphide	260	3600
Diallyl trisulphide	100	-
Dipropyl trisulphide	800	-
Methyl propyl trisulphide	496 ^3^	-
*Phenylpropanoids*		
*trans*-Anethole	3050	>5000
Benzaldehyde	1300	>2000
*trans*-Cinnamaldehyde	2225	12,000
Estragole	1230	5000
Eugenol	3000	2000
*Terpenes*		
*cis*-Ascaridole	4042 ^3^	-
Carvacrol	810	2700
Carvone	3710	4000
Thymol	980	>2000
Geraniol	>4000	>5000

^1^ The experimental values reported for the rat model organism. ^2^ The experimental values reported for the rabbit model organism, except for pesticides and *trans*-cinnamaldehyde, eugenol, and thymol, which were reported for the rat model. ^3^ No data were reported on the selected repositories, so the presented data were predicted using the T.E.S.T. software (version 5.1.2.) through the nearest neighbour method.

**Table 6:** The predicted toxicity classes, hepatotoxicity, neurotoxicity, respiratory toxicity, cardiotoxicity, mutagenicity, developmental toxicity, predicted mutagenicity, and developmental toxicity based on the webtool ProTox 3.0 and the T.E.S.T. software (version 5.1.2.) through the hierarchical clustering method.

Compounds	Prediction Accuracy (%)	Toxicity Class	Hepatotoxicity	Neurotoxicity	Respiratory Toxicity	Cardiotoxicity	Mutagenicity	Developmental Toxicity
*Pesticides*								
Oxamyl	100	1					+	+
Metam sodium	100	3						+
Fluazaindolizine	54	3			+			+
Fluensulfone	23	4						+
Fluopyram	67	4						+
*β-Keto acid/fatty acid derivatives*								
2-Decanone	100	5						+
1-Dodecanol	100	4						
3-Octanol	100	4						+
Octyl acetate	100	5						+
2-Undecanone	100	5						+
*Organosulfur compounds*								
Allicin	54	4						+
Diallyl disulphide	100	3	+					
Diallyl trisulphide	100	3	+					+
Dipropyl trisulphide	100	4						+
Methyl propyl trisulphide	68	3						+
*Phenylpropanoids*								
*trans*-Anethole	100	3		+		+		+
Benzaldehyde	100	2		+		+		
*trans*-Cinnamaldehyde	100	4		+		+		+
Estragole	100	4		+		+		+
Eugenol	100	4		+				+
*Terpenes*								
*cis*-Ascaridole	100	4	+	+	+			+
Carvacrol	100	4		+	+			+
Carvone	100	4		+				+
Thymol	100	4		+	+			+
Geraniol	100	5						

+ predicted positive response.

## Data Availability

The raw data are available from the corresponding author (Jorge M. S. Faria) upon reasonable request.
